# Supplementary material for: Genome-Wide Characterization of Lectin Receptor Kinases in Saccharum spontaneum L. and Their Responses to Stagonospora tainanensis Infection
Source: Plants (Basel). 2021 Feb 8;10(2):322. doi: 10.3390/plants10020322 (PMC7915762; doi:10.3390/plants10020322)
Supplement: Supplementary file 1 [file plants-10-00322-s001.zip › Supplementary Files/Figure S1.pdf]

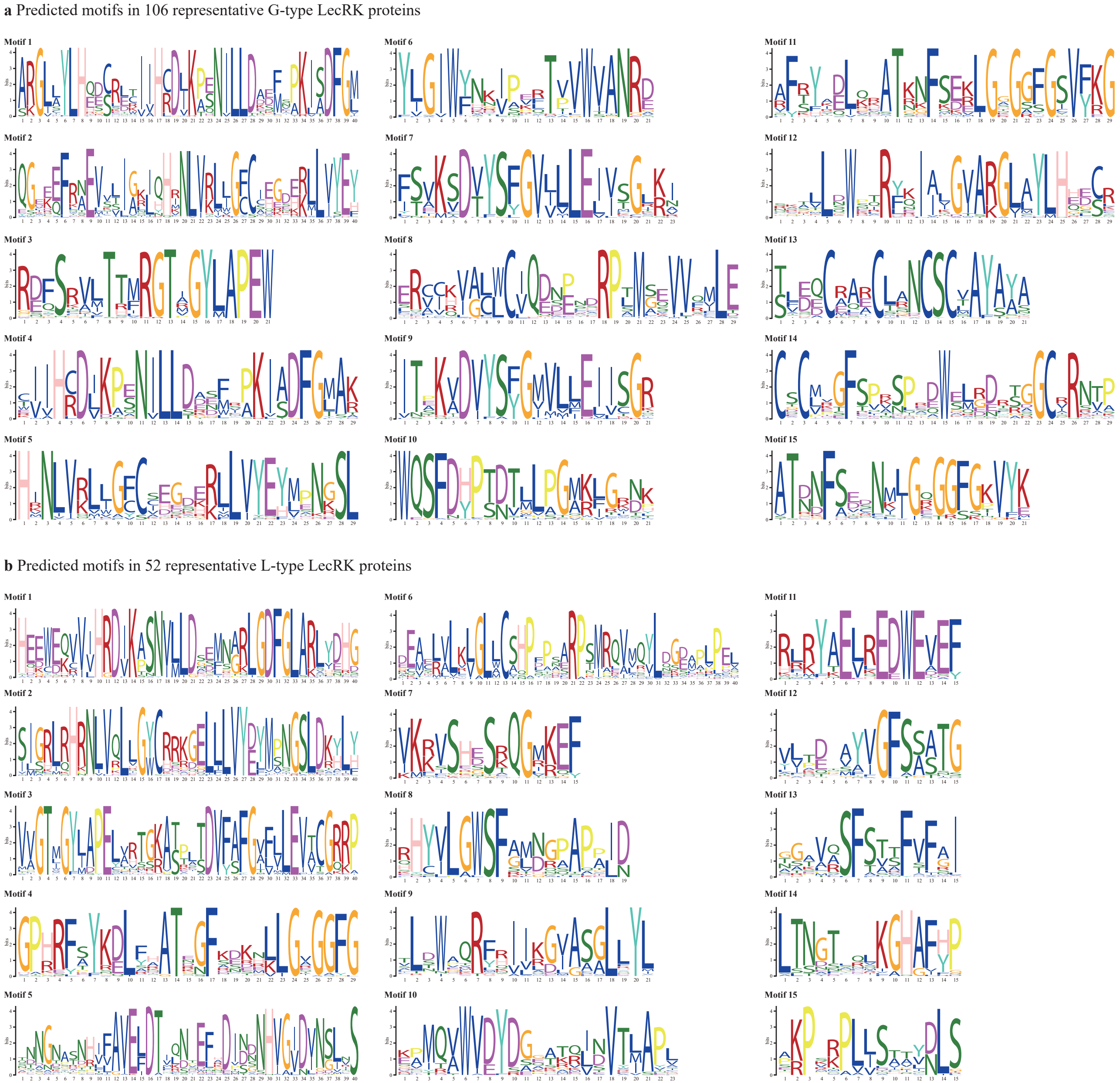

Figure A1. Fifteen distinct conserved motifs of 158 representative non-allelic LecRKs were found. (a) Conserved motifs of 106 representative G-type LecRK proteins. (b) Conserved motifs of 52 representative L-type LecRK proteins.
